# New Record of *Raillietiella furcocerca* (Pentastomida: Raillietiellidae) in *Bothrops atrox* from the Brazilian Amazon: Morphological Characterization and Infection Sites

**DOI:** 10.1007/s11686-026-01435-6

**Published:** 2026-10-26

**Authors:** Anna Célia Oliveira Sarmento, Darlison Chagas-de-Souza, Ricardo Bassini-Silva, Tássio Alves-Coêlho, Síria Ribeiro, Lincoln Lima Corrêa, Alfredo P. Santos-Jr

**Affiliations:** 1 Laboratório de Ecologia e Comportamento Animal, Universidade Federal do Oeste do Pará, Rua Vera Paz, s/n, Salé, Santarém, Pará 68040-255 Brazil; 2 Programa de Pós-Graduação em Biodiversidade, Universidade Federal do Oeste do Pará, Rua Vera Paz, s/n, Salé, Santarém, Pará 68040-255 Brazil; 3 Laboratório Tupiniquim (Parasitologia e Biodiversidade Animal), Avenida Plácido de Castro 1776, Santarém, Pará 68040-090 Brazil; 4 Laboratório de Coleções Zoológicas, Instituto Butantan, São Paulo, SP Brazil; 5 Laboratório de Zoologia e Ecologia Animal, Universidade Federal do Oeste do Pará - Campus de Oriximiná (UFOPA-CORI), Oriximiná, Pará 68270-000 Brazil; 6 Instituto de Ciências e Tecnologia das Águas, Universidade Federal do Oeste do Pará, Rua Vera Paz, Salé, Santarém, Pará 68040-255 Brazil

**Keywords:** Viperidae, Infection site, Host-parasite relationship

## Abstract

**Objective:**

This study aims to document the occurrence of *Raillietiella furcocerca* infecting *Bothrops atrox* in the Lower Amazon region, State of Pará, Brazil, providing morphometric characterization, infection descriptors, and new insights into extrapulmonary infection sites.

**Methods:**

A total of 48 *B. atrox* specimens were examined between 2009 and 2011 (Tapajós National Forest) and 2023–2024 (Santarém and Belterra). Pentastomes were recovered during necropsy, counted, measured, and morphologically identified. Parasitological descriptors (prevalence, mean abundance, and mean intensity) were calculated alongside a literature review of pentastome-snake interactions in the Neotropics.

**Results:**

Nine host specimens were infected (* 9/48*), yielding an overall prevalence of 18.8%, a mean abundance of 0.48, and a mean intensity of 2.56. Pentastomes were recovered from both the lungs and the coelomic cavity, actively attached to visceral serosa and associated with focal tissue lesions.

**Conclusion:**

This study provides the first quantitative parasitological baseline for *R. furcocerca* in *B. atrox* from the Amazonian biome and confirms the coelomic cavity as a viable antemortem extrapulmonary infection site and provide update literature review and distribution mapping on pentastomids infection in Neotropical snakes.

## Introduction

Pentastomida is a group of worm-like endoparasites belonging to Pancrustaces, class Ichthyostraca (i.e., Branchiura + Pentastomida), that mainly parasitize the respiratory system of vertebrates [1–5]. Approximately 144 valid species have been recognized in the literature, characterized by an elongated, pseudo-segmented body, a subterminal or central mouth, and two pairs of sclerotized anterior hooks that aid in attachment to the host tissues [1, 3, 4] The adults are obligate hematophages, and their life cycle may involve intermediate hosts, usually insects, before reaching the reproductive stage in the respiratory tract of the definitive hosts [6–8].

**Fig. 1:**
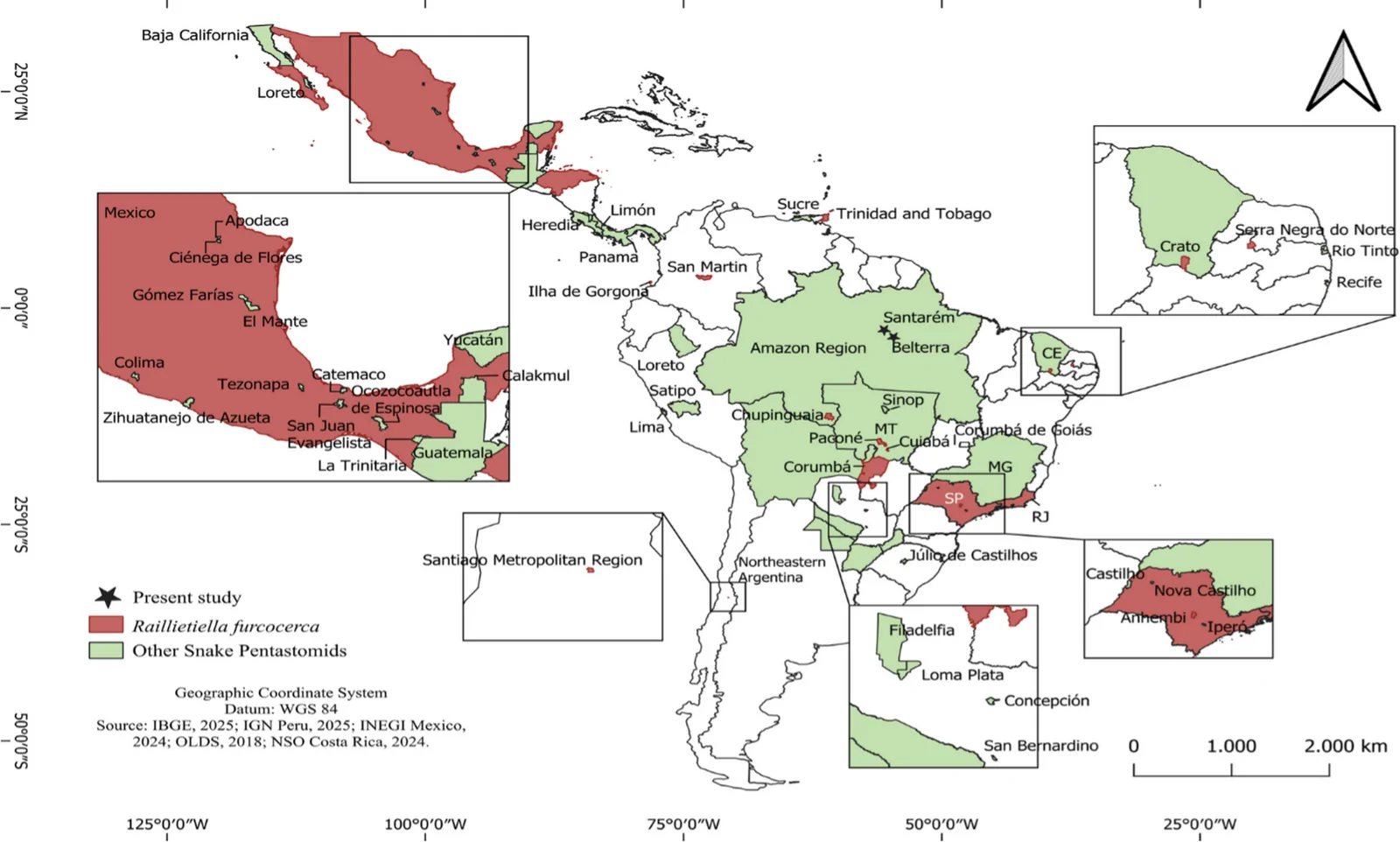
Map of occurrence records for *Raillietiella furcocerca* and other snakes pentastomids in the Neotropical region. Note: Some records lack precise locality data. In these cases, we included generalized areas (e.g., Amazon Region)

In the Neotropical region, pentastomids research has a comprehensive history spanning diverse ecosystems in Argentina, Peru, Chile, Costa Rica, Mexico and Brazil [9–13]. The family Raillietiellidae Sambon, 1922, represented mainly by the genus *Raillietiella* Sambon, 1910 and comprises a widespread lineage that utilizes insects as intermediate hosts and develops in the lung tissues of reptiles, particularly lizards and snakes [3, 9–16]. The species *Raillietiella furcocerca* (Diesing, 1836) was originally described as *Pentastoma furcocerca* Diesing, 1836 from a boid snake, *Boa constrictor* Linnaeus, 1758 collected in Brazil. However, its taxonomic history was subsequently refined by Heymons (1935) and Rêgo (1983, 1984), with *Raillietiella gomesi* Motta, 1963 later recognized as a junior synonym following detailed morphometric analyses of hook architecture and spicules. Despite its recognized status as a widespread euryxenous parasite infecting multiple snake families (Boidae, Colubridae, Dipsadidae and Viperidae), the vast majority of South American records originate from the Atlantic Forest, Caatinga and Cerrado biomes, leaving major biogeographic gaps across northern Amazonian ecosystems (Fig. 1) [6, 14–18].

In Brazil, six species of genus *Raillietiella* (Raillietiellidae) have been recorded in snakes [3, 9–13]. Despite the relatively large number of studies involving pentastomids of the genus *Raillietiella* in Neotropical snakes [9, 10, 14–16], records of secondary infection sites outside the host’s respiratory system are scarce [12, 17], with no reports of the presence of adult pentastomids in other infection sites in their hosts.

In this study, we report the occurrence of *R. furcocerca* parasitizing the Amazonian lancehead *Bothrops atrox* (Linnaeus, 1758) from the Brazilian Amazon, state of Pará, Brazil. We provide morphological and morphometric characterization, quantifying infection parameters (prevalence, mean intensity and mean abundance) and information on extrapulmonary infection sites. Additionally, we present an updated literature review and distribution mapping on pentastomid infection in snakes across the Neotropical region, compiling historical and current data on the occurrence and geographical distribution of these parasites.

## Materials and Methods

### Study Area and Snake Capture

A total of 48 specimens of *B. atrox* were collected from six sampling sites across the municipalities of Belterra and Santarém, State of Pará, Brazil (Fig. 1; Table 1). Snake taxonomy followed Costa et al. (2026). Sampling was conducted during two distinct periods: December 2009 to December 2011 (* n=37*) and December 2023 to July 2024 (* n=11*). Collected specimens were anesthetized and euthanized using barbiturates in accordance with the guidelines of the Conselho Nacional de Controle de Experimentação Animal (CONCEA).

After parasitological analyses, the snakes were fixed in 10% formalin, preserved in 70% ethanol, and deposited in the Coleção Herpetológica do Laboratório de Ecologia e Comportamento Animal (LECAn) of the UFOPA and in the Linha de Pesquisa em Herpetologia da Amazônia (LPHA), of the Laboratório de Pesquisas Zoológicas (LPZ) of the Universidade da Amazônia (UNAMA).

### Parasite Collection and Preparation

Live snakes collected in the field were transported directly to the laboratory in suitable containers. The procedures of euthanasia were performed at the laboratory using barbiturates in strict accordance with ethical guidelines. Immediately post-mortem, full necropsy and parasitological examination were carried out to prevent tissue decay or passive post-mortem displacement of parasites. The liver, lungs, heart, oral cavity, larynx, stomach, small and large intestines, coelomic cavity, and kidneys were thoroughly examined in situ for the presence of pentastomes. The parasites found were collected and subsequently stored in microtubes with 70% ethanol for morphological identification. While pentastomes were the primary target of this investigation, other parasitic taxa, specifically helminths including nematodes and cestodas, were also recovered during necropsies. These non-pentastome parasites were fixed, quantified, and stored separately in 70% ethanol for dedicated taxonomic analyses in forthcoming studies.

For morphological identification, the specimens were clarified in Hoyer’s medium, temporarily mounted on slides, and preserved in 70% ethanol and identified according to the literature [6, 19–22]. Subsequently, they were preserved in ethanol and prepared as temporary slides for analysis by light microscopy at magnifications of 100 to 400x. Photomicrographs were processed using a Zeiss Axioplan microscope with an Axiocam ERc 5s camera.

Morphometric traits, including total body length and width, dimensions and structural characteristics of sclerotized hooks and male copulatory spicules, as well as the number of superficial body annulations, were measured using Zen Blue Edition 2 software. Following morphometric analysis, specimens were unmounted, rinsed, and preserved in 70% ethanol for long-term storage in the institutional parasitological collection.

### Scanning Electron Microscopy (SEM) Characterization

Specimen preparation for SEM was performed following adapted standard protocols [23]. Parasite samples previously stored in 70% ethanol were post-fixed in 2.5% glutaraldehyde in 0.15 M phosphate buffer (pH 7.3) for 24 h and subsequently fixed in 1% osmium tetroxide in the same buffer for 2 h. Following fixation, samples were washed in phosphate buffer and dehydrated through an ascending ethanol series (50%, 70%, 80%, 90%, and 100%). Critical-point drying was performed using a CPD 020 dryer (Balzer Union) with liquid CO₂. The dried specimens were mounted on aluminum stubs with double-sided carbon tape, sputter-coated with a gold-palladium layer, and examined using a Leo Stereoscan S-440 scanning electron microscope.

### Data Analysis

Ecological terms and parasitological descriptors, including prevalence, mean abundance, and mean intensity of infection, were calculated following Bush et al. (1997) [24]. Descriptors and their corresponding 95% confidence intervals (95% CI) were estimated using Quantitative Parasitology 3.0 software according to Reiczigel et al. (2019) [25]. The 95% CI for prevalence was calculated using the exact Clopper–Pearson method, whereas 95% CIs for mean abundance and mean intensity were derived using bootstrap resampling procedures with 2,000 interations to account for aggregated parasite distributions and sample size. To explore morphometric similarities among male and female specimens from the present study and compiled literature records, a cluster analysis based on the Bray–Curtis dissimilarity index was performed using PAST 4.0 software. The input data matrix comprised multivariate morphometric profiles, where rows represented each host/study source and columns corresponded to mean values of measured anatomical structures. Because measured structures differed substantially in scale, data were standardized (* z*-score normalization) prior to analysis to prevent larger structures from disproportionately influencing dissimilarity distances. Missing values in the literature dataset were handled using pairwise deletion.

### Ethical Approval

Host capture was authorized by the Biodiversity Authorization and Information System (SISBIO) under numbers 77466/6 and 14018-8, and the procedures were in accordance with the CONCEA, approved by the Comitê de Ética no Uso Animal of the Universidade Federal do Amapá (UNIFAP), certificate 011/2021, and registered with the Sistema Nacional de Gestão do Patrimônio Genético e do Conhecimento Tradicional Associado of the Brazilian government under number AC5C1B1. The slides were deposited in the Parasitological Collection of the LECAn of the UFOPA under voucher numbers [UFOPA-P (Pen 01)] and [UFOPA-P (Pen 02)].

### Literature Review Compilation

A literature review and compilation of pentastome infection records in Neotropical snakes were conducted in accordance with the Preferred Reporting Items for Systematic Reviews and Meta-Analyses (PRISMA) guidelines [26, 27]. The literature search was performed in March 2026 across four electronic databases: Web of Science, Scopus, SciELO (Scientific Electronic Library Online), and ScienceDirect. The search strategy utilized combinations of Boolean operators (AND / OR) with the following core keywords: (“snake” OR “reptilia”) AND (“parasitic pentastomids” OR “pentastomida” OR “tongue worms”) AND (“Brazil” OR “neotropical”). Full search strings tailored to each specific database were applied without date restrictions. Theses, dissertations, and conference proceedings abstracts were excluded.

All retrieved articles were imported into the web platform Rayyan [28], where automated and manual removal of duplicate records was performed. Title and abstract screening were conducted directly within the platform in an independent and blind manner by two reviewers (ACOS and SR) according to the eligibility criteria. Any evaluation conflicts were subsequently resolved through consensus discussions among the authors. Peer-reviewed original articles reporting pentastome interactions with snakes in the Neotropical region were included. Data from full-text eligible studies were extracted into Microsoft Excel^®^ spreadsheets, detailing: authors, publication year, study location (state, region, coordinates where available), host species, pentastome species, site of infection, and parasitological metrics. The entire selection process was organized following the PRISMA flow diagram guidelines.

## Results

The specimens captured of *B. atrox* (28 females and 20 males) were examined across two sampling periods (2009–2011 and 2023–2024). Overall, nine hosts were parasitized by pentastomes, yielding an overall prevalence of 18.8% (9.0–32.6%). A total of 23 specimens of *R. furcocerca* were recovered from the lungs and coelomic cavity, resulting in an overall mean abundance of 0.48 (0.15–1.10) and an overall mean intensity of 2.56 (1.33–4.78).

Sampling comparison revealed a markedly higher infection rate in the second sampling period (2023–2024; 54.5%, 6/11) compared to the earlier period (2009–2011; 8.1%, 3/37), with a corresponding increase in mean infection intensity (3.33 vs. 1.00 parasite/infected host, respectively). Detailed period-specific parasitological descriptors and their 95% confidence intervals are presented in Table 2.

### Literature Review

The systematic search review 53 publications reporting pentastomids infections in Neotropical snakes, yielding a total of 132 host-parasite interaction records (defined here as each unique occurrence of a pentastomids species/genus associated with a snake species in each study). These records encompass 9 pentastomid species, in addition to records identified only at the genus level or as Pentastomida indet., distributed across 5 genera, 4 families, and 2 orders, infecting 59 snake species belonging 5 families (Boidae, Colubridae, Dipsadidae, Elapidae and Viperidae). High representation was observed for two families belonging to distinct orders: was observed for Porocephalidae Fain, 1961 (Order Porocephalida; *n* = 57 records) and Raillietiellidae (Order Raillietiellida: *n* = 35 records), alongside Sebekidae Heymons, 1922 (Order Porocephalida; *n* = 14 records) and Cephalobaenidae Heymons, 1922 (Order Raillietiellida; *n* = 14 records). Among the five pentastomid genera reported, *Porocephalus* Humboldt, 1811 exhibited the highest record frequency (*n* = 41), followed by *Raillietiella* Sambon, 1910 (*n* = 35), *Kiricephalus* Sambon, 1922 (*n* = 17), *Cephalobaena* Heymons, 1922 (*n* = 14), *Sebekia* Sambon, 1922 (*n* = 14).At the species level, all 9 identified pentastomid species were: *Raillietiella furcocerca* (Diesing, 1836) (*n* = 29 records), *Porocephalus crotali* (Humboldt, 1808) (*n* = 19), *Cephalobaena tetrapoda* Heymons, 1922 (*n* = 14), *Kiricephalus coarctatus* (Diesing, 1850) (*n* = 12), *Porocephalus stilesi* Sambon, 1922 (*n* = 10), *Sebekia oxycephala* (Diesing, 1836) (*n* = 9), *Porocephalus clavatus* (Wyman, 1845) (*n* = 5), and *Raillietiella venteli* Motta, 1965 (*n* = 2). The geographic distribution of these records was predominantly centered in Brazil, with additional occurrences reported from Argentina, Peru, Chile, Colombia, Venezuela, Paraguay, French Guiana, Costa Rica, Mexico, Panama, Honduras, Bolivia, Guatemala and Trinidad (Table 3).

Infection site analysis indicated the respiratory system (lungs) as the primary site of infection for *Raillietiella furcocerca*. However, infections in extrapulmonary sites, such as the stomach, liver, intestine, mesentery and coelomic cavity had been previously documented in the literature for other Neotropical hosts including *Boa constrictor*, *Lachesis muta* (Linnaeus, 1766), and *Crotalus durissus* Linnaeus, 1758 (e.g., Rego, 1983; Almeida et al., 2008). The occurrence of *R. furcocerca* in the gastrointestinal tract of *Crotalus durissus terrificus* (Laurenti, 1768) supports our findings of coelomic infection in *Bothrops atrox*, reinforcing that pentastomids may migrate to extrapulmonary visceral sites during high-intensity infections or late developmental stages (Table 3).

### Taxonomic Summary

*Raillietiella furcocerca* (Diesing, 1836) Sambon, 1910.

**Synonym**: *Raillietiella gomesi* Motta, 1963.

**Phylum**: Arthropoda von Siebold, 1848.

**Superclass**: Oligostraca Zrzavý, Hypša & Vlášková, 1997.

**Class**: Ichthyostraca Zrzavý, Hypša & Vlášková, 1997.

**Subclass**: Pentastomida Diesing, 1836.

**Order**: Raillietiellida Almeida & Christoffersen, 1999.

**Family**: Raillietiellidae Sambon, 1922.

**Genus**: *Raillietiella* Sambon, 1910.

**Type host**: *Boa constrictor* Linnaeus, 1758.

**Type locality**: Mato Grosso State, Brazil.

**Additional host**:

Viperidae: *Bothrops atrox*, *Bothrops jararaca* (Wied-Neuwied, 1824), *Crotalus durissus* Linnaeus, 1758.

Boidae: *Boa constrictor*, *Epicrates cenchria* (Linnaeus, 1758), *Eunectes murinus* (Linnaeus, 1758), *Corallus hortulana* (Linnaeus, 1758).

Colubridae: *Philodryas nattereri* (Steindachner, 1870), *Philodryas olfersii* (Lichtenstein, 1823), *Leptodeira annulata* (Linnaeus, 1758), *Clelia Clelia* (Daudin, 1803), *Oxyrhopus trigeminus* Duméril, Bibron & Duméril, 1854, *Spilotes pullatus* (Linnaeus, 1758), *Erythrolamprus poecilogyrus* (Wied-Neuwied, 1824), *Chironius* spp.

**Additional locality**: Neotropical Region. South America: Brazil (Amazonia, Caatinga, Cerrado, and Atlantic Forest biomes—States of Pará, Ceará, Pernambuco, Bahia, Minas Gerais, Rio de Janeiro, São Paulo, Mato Grosso), Argentina (Chaco biome), Colombia, and Venezuela.

**Infection site**: Respiratory tract (lungs, trachea; primary site) and extra-pulmonary visceral sites (coelomic cavity, visceral serosa; present study and literature).

**Prevalence in *Bothrops atrox* (present study)**: 18.8% (9.0–32.6).

**Distribution in present study**: Tapajós National Forest (3º53′241′′S 55º05′224′′W), municipality of Belterra (2°38′18′′S 54°56′41′′W); and Vale dos Oliveiras, municipality of Santarém (2°32′51′′S 54°53′53′′W), State of Pará, Brazil.

#### Morphological Characteristics

**Fig. 2:**
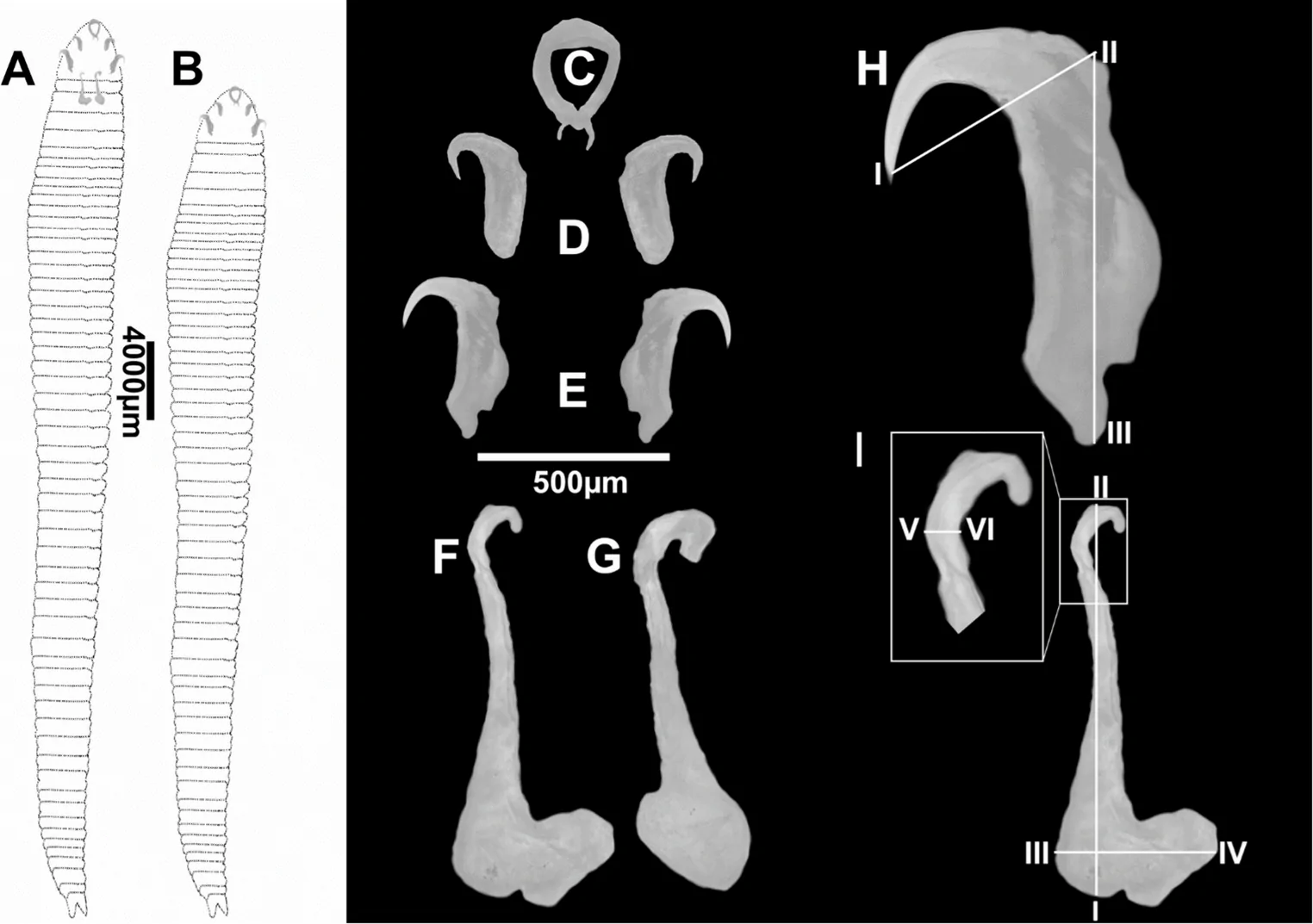
*Raillietiella furcocerca* found in *Bothrops atrox* in the State of Pará, Brazil. **A** Male specimen, **B** female specimen, **C** oral complex, **D** pair of frontal hooks, **E** pair of posterior hooks, **F** right copulatory dilator, **G** left copulatory dilator, **H** hook measurement template (blade measurements = I–II; total hook length = I–II–III), and **I** copulatory dilator measurement template

**Fig. 3:**
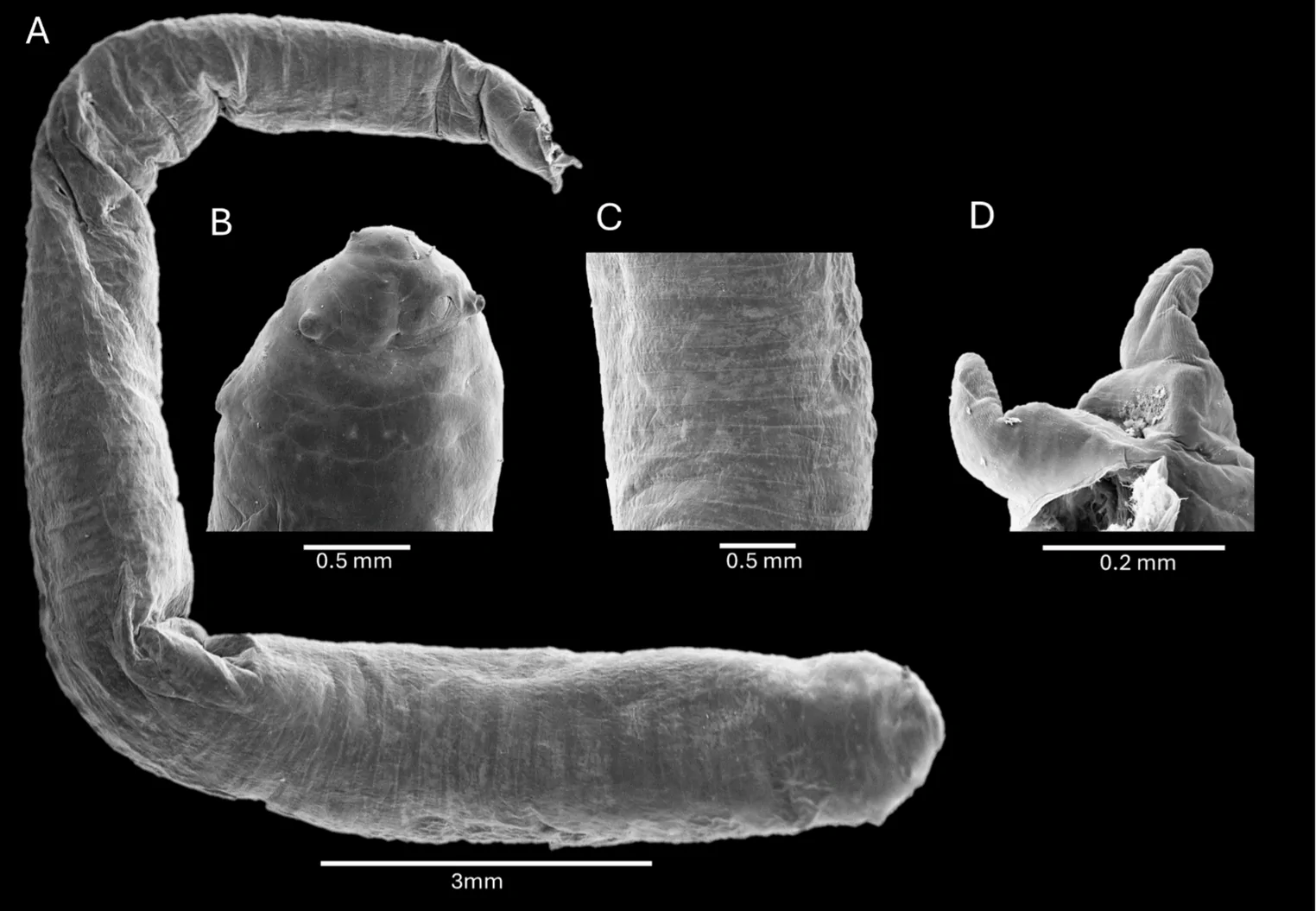
Male of *Raillietiella furcocerca*. **A** General view of the parasite, **B** cephalothorax, **C** annulation in the median region of the body, and **D** terminal portion of the tail

The analyzed specimens present a cylindrical, vermiform body flattened in both sexes (Figs. 2A and 3A), with variable dimensions and annulations that are difficult to observe and definitively quantify (Fig. 3C) (see Table 4). In male specimens, a rounded contour is observed in the cephalothorax, an anterior oral cavity (Fig. 2C), and a trapezoidal arrangement of hooks. In male individuals, there are internal copulatory dilators that are elongated with a C-shaped curvature at the distal position and broad bases with ridges (Figs. 2A, F, G). It is possible to observe shortened parapoids reduced to three protuberances in the hooks, a chitinous accessory structure is noted (Fig. 3B). Both pairs of hooks have a developed base, with the posterior hooks being larger than the anterior ones (Fig. 2D, E). The copulatory dilators, observed only in males, are visibly elongated, with an extended base and the presence of a crest, being slightly positively curved at the distal part of the organ.

**Fig. 4:**
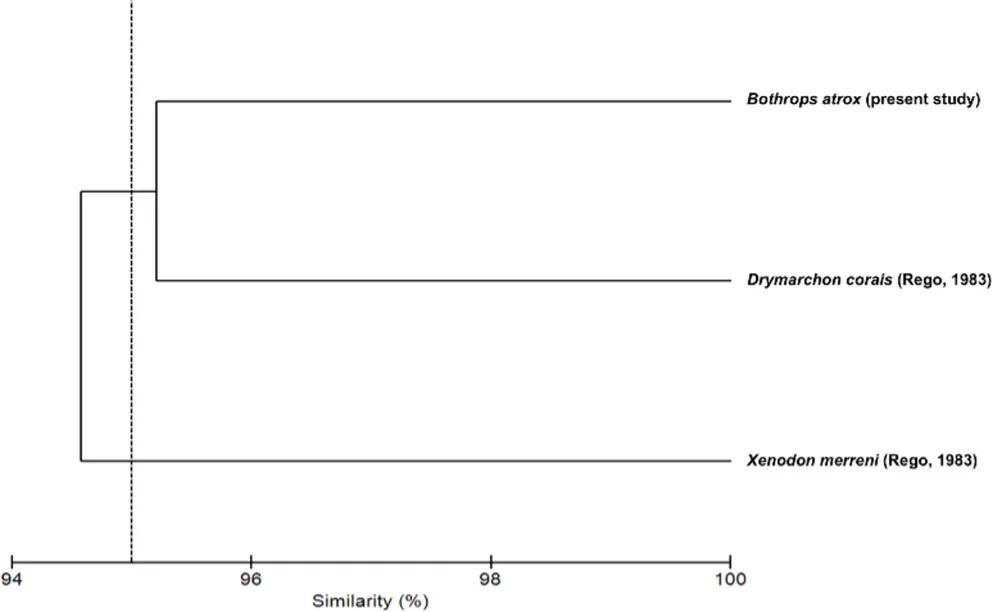
Similarity dendrogram for male *Raillietiella furcocerca* compared with host snakes previously reported in the literature

**Fig. 5:**
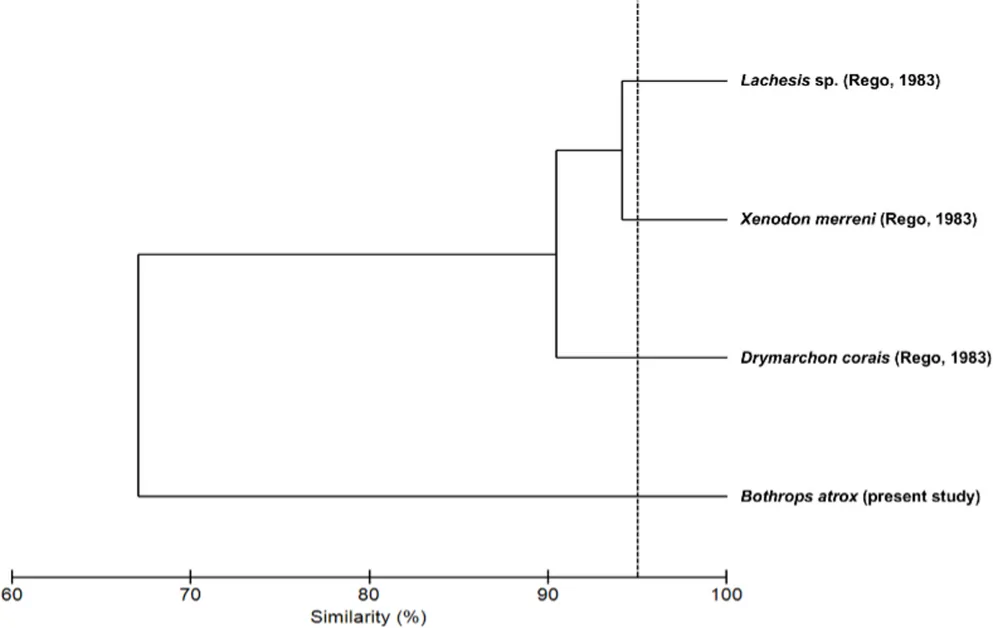
Similarity dendrogram for female *Raillietiella furcocerca* compared with host snakes previously reported in the literature

Female specimens may present varied dimensions. However, the buccal complex, as in males, shows a more chitinous ring-shaped region, with a bifurcated termination. The arrangement and proportion of hooks are equivalent in both sexes. Females have cephalothorax structures comparable to those of males but lack copulatory dilators. Using similarity dendrograms and mean values from regions where *R. furcocerca* was sampled, we observed that the males in our study share over 95% morphometric similarity with *R. furcocerca* specimens found in *Drymarchon corais* (Boie, 1827) in the Brazilian states of São Paulo and Mato Grosso [29] (Fig. 4). However, when we compared the morphometric similarity among female *R. furcocerca* specimens in this study, we observed low similarity to other specimens found in previously reported hosts. This demonstrates that variation in parasite size may occur due to various factors, not merely because they were found in different hosts (Fig. 5).

#### Infection Sites

**Fig. 6:**
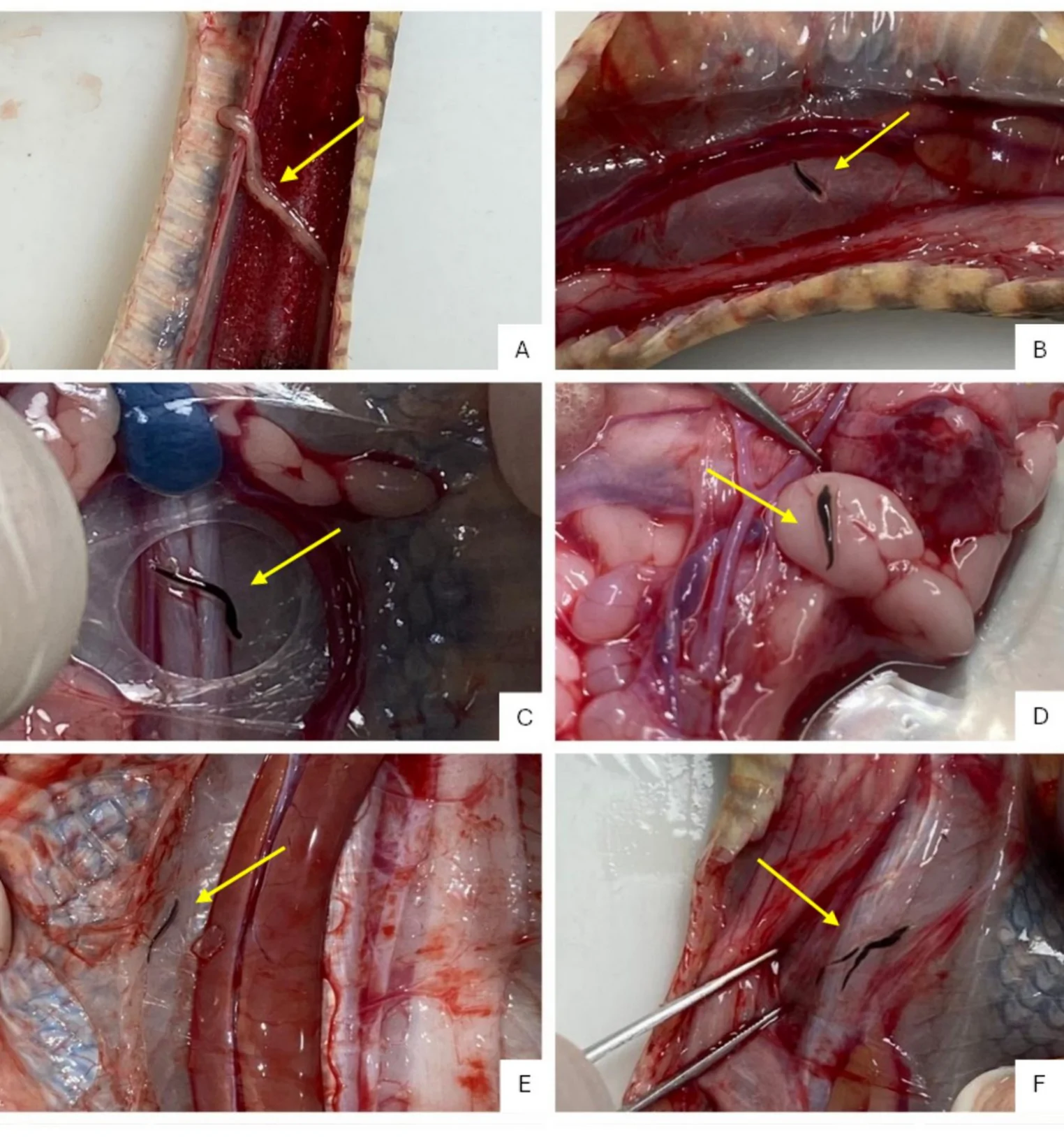
*Raillietiella furcocerca* specimens found in *Bothrops atrox*. **A** and **B** pentastomid visible in the lung and air sac, and **C**–**F** pentastomids found in the gastrointestinal cavity of the host

**Fig. 7:**
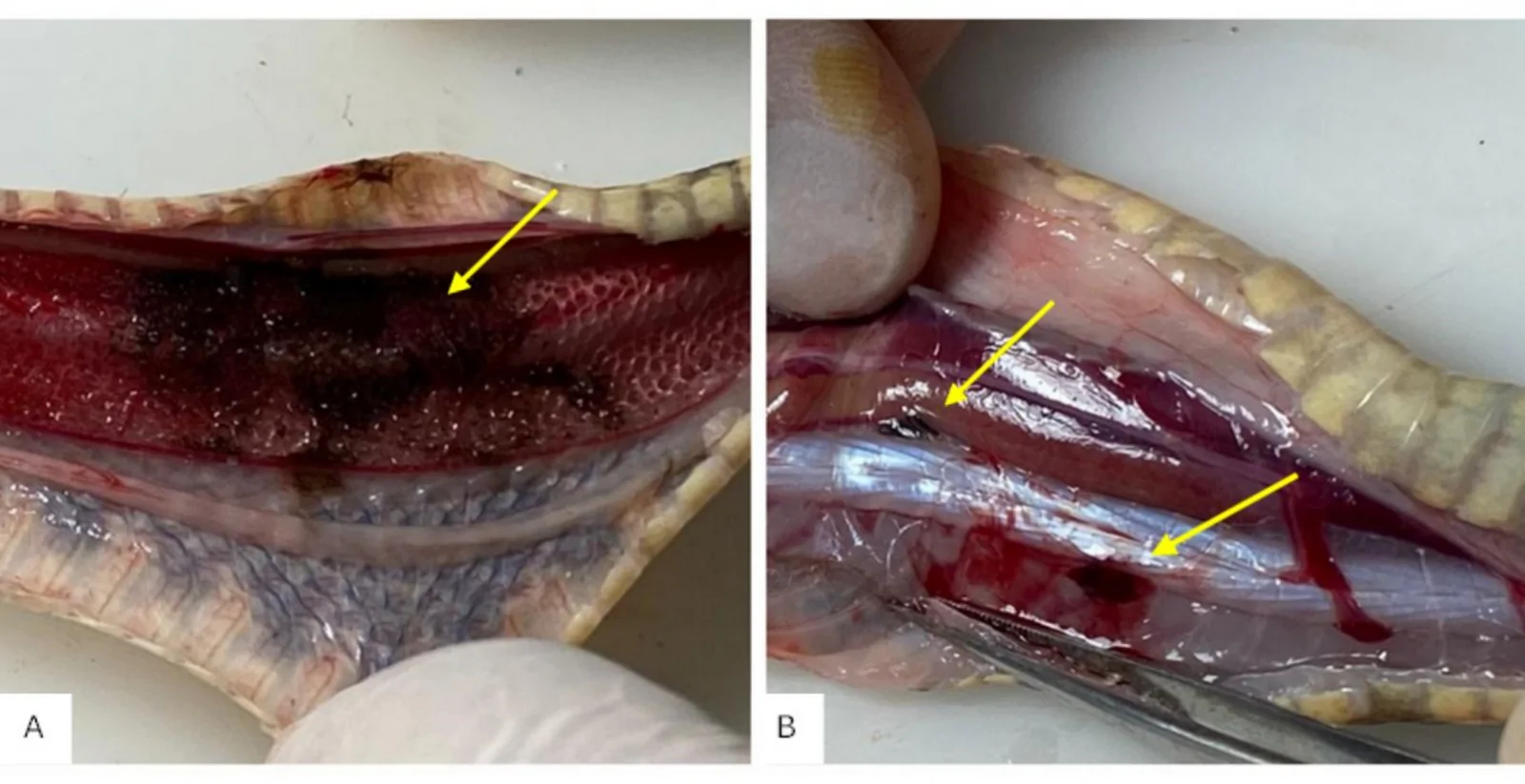
Lesions caused by *Raillietiella furcocerca* in *Bothrops atrox* specimens. **A** Lesions in lung tissue and **B** in the coelomic cavity

Pentastomids were recovered from both the lungs and the coelomic cavity (near the liver, small intestine, large intestine, and cloacal adipose tissue) (Fig. 6). Specimen recovery in the coelomic cavity revealed parasites actively attached to the visceral tissues, directly associated with localized hemorrhages and focal tissue reactions, supporting their classification as true extrapulmonary infection sites rather than post-mortem displacement. In addition, macro-lesions were identified in the pulmonary tissue, characterized by focal parenchymal damage and blood extravasation into the lung lumen (Fig. 7).

**Table 1:** List of locations and collection periods for *Bothrops atrox* specimens in two municipalities in the state of Pará, Brazil

Site	Location	Municipality	Coordinates	Collection period	Specimens (*n*)
1	FLONA Tapajós (BR-163, km 83)	Belterra	03°03′ S 054°57′ W	Dec 2009–Dec 2011	37
2	Urban area	Belterra	2°38′ 18′′ S 54°56′ 41′′ W	Dec 2023–Jul 2024	6
3	Ramal do Jatobá	Santarém	2°32′35′′ S 54°51′47′′ W	Dec 2023–Jul 2024	1
4	Vale dos Oliveiras	Santarém	2°32′ 51′′ S 54°53′53′′ W	Dec 2023–Jul 2024	2
5	Lago do Mapiri	Santarém	2º42′82′′ S 54º74′70′′ W	Dec 2023–Jul 2024	1
6	Tapajós Campus (UFOPA)	Santarém	2°25′13′′ S 54°44′26′′ W	Dec 2023–Jul 2024	1
Total					48

**Table 2:** Prevalence, mean abundance, and mean intensity of *Raillietiella furcocerca* in *Bothrops atrox* collected during two sampling periods in the Brazilian Amazon. C.I: Confidence interval

Period	Hosts infected/examined	Number of parasites	Prevalence (%)	Mean abundance	Mean intensity
2009–2011	3/37	3	5.4 (C.I. 0.6–18)	0,08 (C.I. 0.02–0.27)	1,5 (C.I. 1.0–2.0)
2023–2024	6/11	20	45,5 (C.I. 16.0–74.9)	1,82 (C.I. 0.0–3.98)	4,0 (C.I. 0.0–3.98)
Total	9/48	23	18.8 (9.0–32.6)	–	–

**Table 3:** List of records of pentastomids parasitic in neotropical snakes. NI = not informed

Family/species hosts	Pentastomids species	Site of infection (when informed)	Location	References
**Boidae**				
*Boa constrictor*	*Porocephalus crotali*,* Sebekia sp.*	Lung and trachea	Cuiabá, Mato Grosso, Brazil	[30]
*B. constrictor*	*Porocephalus crotali*	Lung and coelomic cavity	Brazil	[30]
*B. constrictor*	*Raillietiella furcocerca*	NI	São Paulo e Rio de Janeiro, Brazil	[31]
*B. constrictor*	*Porocephalus* sp.	Lung	Recife, Pernambuco, Brazil	[32]
*B. constrictor*	*Porocephalus clavatus*	Lung	Departamento Loreto, Peru	[5]
*B. constrictor*	*Sebekia oxycephala*	Lung	Poconé, Brazil	[33]
*B. constrictor*	*Porocephalus* sp.	Coelomic cavity	Brazil	[10]
*Boa constrictor occidentalis* (Phillippi, 1873)	Pentastomida indet.^a^	Lung	Northeastern Argentina	[34]
*Boa imperator* Daudin, 1803	*Porocephalus clavatus*	NI	La Fortuna, Limón, Costa Rica	[35]
*B. imperator*	*Raillietiella furcocerca*	Lung	South America	[36]
*B. imperator*	Porocephalus clavatus*	NI	South America	[37]
*Epicrates cenchria* (Linnaeus, 1758)	Pentastomida indet.^a^	Lung	Northeastern Argentina	[34]
*E. cenchria*	*Porocephalus clavatus*	Lung	Lima, Peru	[5]
*Eunectes murinus* (Linnaeus, 1758)	*Porocephalus clavatus*	Lung	Rio Araguaia, Brasil	[30]
*E. murinus*	*Sebekia oxycephala* (nymph)*	Encysted in the mesentery and viscera	Mato Grosso, Brazil	[30]
*E.murinus*	*Sebekia oxycephala*	Lung	Poconé, Brazil	[33]
*Eunectes notaeus* Cope, 1862	Pentastomida indet.^a^	Lung	Northeastern Argentina	[34]
**Colubridae**				
Colubridae sp. indet.	*Raillietiella furcocerca*	Blood vessels/cyst	NI	[30]
*Drymarchon corais* Stejneger 1899	*Raillietiella furcocerca*	Coelomic cavity	Cuiabá, Mato Grosso, Brazil	[30]
*D.corais*	*Raillietiella furcocerca*, *Kiricephalus coarctatus*	NI	Instituto Butantan, São Paulo, Brazil	[12]
*D.corais*	*Raillietiella furcocerca*	NI	Mexico	[38]
*Chironius carinatus* Linnaeus, 1758	*Kiricephalus coarctatus*	NI	Amazônia, Brazil	[31]
*Chironius grandisquamis* (Peters, 1869)	*Kiricephalus coarctatus*	Lung, trachea and cavity	Parque Nacional Omar Torrijos, Panamá	[39]
*Hydrodynastes bicinctus* (Hermann, 1804)	*Kiricephalus coarctatus*	Lung and body cavity (nymphs)	Approuague River, French Guiana	[12]
*Hydrodynastes gigas* (Duméril, Bibron & Duméril, 1854*)*	*Porocephalus crotali*	Lung	Chaco, Argentina	[40]
*Leptodeira annulata* (Linnaeus, 1758)	*Raillietiella furcocerca*	Lung	Ceará, Brazil	[14]
*Leptophis ahaetulla* (Linnaeus, 1758)	*Kiricephalus coarctatus*	Lung, trachea and coelomic cavity	Parque Nacional Omar Torrijos, Panamá	[39]
*Leptophis liocercus* (Wied, 1824)	*Cephalobaena tetrapoda*	NI	Misiones, Loreto, Argentina	[41]
*Mastigodryas bifossatus* (Raddi, 1820)	*Raillietiella furcocerca*	Coelomic cavity	Cuiabá, Mato Grosso, Brazil	[30]
*M. bifossatus*	*Kiricephalus coarctatus*	Coelomic cavity	Brazil	[42]
*M. bifossatus*	Pentastomida indet.^a^	Lung	Northeastern Argentina	[34]
*Mastigodryas boddaerti* (Sentzen, 1796)	*Kiricephalus* sp.	NI	Trinidad, West Indies	[43]
*M. boddaerti*	*Raillietiella furcocerca*	NI	Ilha de Gorgona, Colômbia	[38]
*Mesotes strigatus* (Günter, 1858)	Pentastomida indet.^a^	Lung	Northeastern Argentina	[34]
*Oxybelis aeneus* (Wagler, 1824)	*Cephalobaena tetrapoda*	Lung	Serra Negra do Norte, Rio Grande do Norte, Brazil	[21]
*Pseudoboa nigra* (Duméril, Bibron & Duméril, 1854)	*Raillietiella furcocerca*	Nasopharynx	Chupinguaia, Rondônia, Brazil	[44]
*Palusophis bifossatus* (Raddi, 1820)	*Raillietiella furcocerca*, *Kiricephalus coarctatus*	NI	São Paulo e Rio Janeiro, Brazil	[31]
*Spilotes* sp.	*Raillietiella furcocerca*	Mesentery	Cuiabá, Mato Grosso, Brazil	[30]
*Spilotes pullatus (Linnaeus*,* 1758)*	*Porocephalus crotali*, *Sebekia sp.*	Lung	Fazenda Real de Ipanema, Iperó, São Paulo, Brazil	[30]
**Dipsadidae**				
Natricidae / Dipsadidae sp. indet.	*Raillietiella furcocerca* (nymph)*	NI	NI	[30]
*Conophis lineatus* Duméril, Bibron & Duméril, 1854	*Raillietiella furcocerca*	NI	Honduras	[38]
*Clelia clelia* (Daudin, 1803)	*Raillietiella furcocerca*	Distal membranes of lung	Barbascal, Meta, Colômbia	[45]
*C. clelia*	*Kiricephalus* sp.	Immature nymph	Trinidad, West Indies	[43]
*C. clelia*	Pentastomida indet.^a^	Lung	Northeastern Argentina	[34]
*Erythrolamprus aesculapii* (Linnaeus, 1758)	*Kiricephalus coarctatus*	Nymphs	Bolívia	[43]
*Erythrolamprus bizona* Jan, 1863	*Kiricephalus coarctatus*	Nymphs	Panamá	[43]
*Erythrolamprus poecilogyrus* (Wied-Neuwied, 1824)	*Raillietiella furcocerca*	Lung	Loma Plata, Paraguay	[46]
*E. poecilogyrus*	*Raillietiella* sp.	Lung	Crato, Ceará, Brazil	[10]
*E. poecilogyrus*	*Kiricephalus cf. coarctatus*	Lung	Route 2, Pilcomayo, Northeastern Argentina	[47]
*Erythrolamprus almadensis* (Wagler, 1824)	*Raillietiella furcocerca*	Lung, nymphs in intestinal epithelium	Loma Plata, Paraguay	[46]
*Helicops angulatus* (Linnaeus, 1758)	*Porocephalus stilesi*	Lung	South America	[41]
*H. angulatus*	*Sebekia oxycephala*	Nymphs in the stomach and intestine	Rio Tinto, Paraíba, Brazil	[11]
*Helicops carinicaudus* (Wied-Neuwied, 1824)	Pentastomida indet.^a^	Lung	Northeastern Argentina	[34]
*Helicops infrataeniatus* Jan, 1865	*Sebekia oxycephala*	Coelomic cavity	Castilho, São Paulo, Brazil	[17]
*Helicops leopardinus* (Schlegel, 1837)	*Sebekia oxycephala*	NI	Poconé, Brazil	[29]
*Lygophis lineatus* (Linnaeus, 1758)	*Cephalobaena tetrapoda*	Lung	Crato, Ceará, Brazil	[22]
*Philodryas baroni* (Berg, 1895)	*Cephalobaena tetrapoda*	Lung and trachea	Filadelfia, Paraguay	[48]
*P. baroni*	Pentastomida indet.^a^	Lung	Northeastern Argentina	[34]
*Philodryas chamissonis* (Wiegmann, 1835)	*Raillietiella* sp.	Lung	Santiago, Chile	[49]
*Philodryas nattereri* (Steindachner, 1870)	*Cephalobaena tetrapoda*,* Raillietiella furcocerca*	Lung	Serra Negra do Norte, Rio Grande do Norte, Brazil	[9]
*P. nattereri*	*Cephalobaena tetrapoda*,* Raillietiella furcocerca*	Lung	Crato, Ceará, Brazil	[9]
*Philodryas olfersii* (Lichtenstein, 1823)	*Cephalobaena tetrapoda*,* Raillietiella furcocerca*	Lung	Ilha Grande, Delta do Parnaíba, Piauí, Brazil	[15]
*Philodryas patagoniensis* (Girard, 1858)	*Cephalobaena tetrapoda*	Lung, trachea and air sac	Concepción, Paraguay	[46]
*P. patagoniensis*	Pentastomida indet.^a^	Lung	Northeastern Argentina	[34]
*P. patagoniensis*	*Raillietiella furcocerca*	Lung and trachea	Loma Plata, Paraguay	[46]
*P. patagoniensis*	*Cephalobaena tetrapoda*,* Raillietiella furcocerca*	Lung	Corrientes, Argentina	[50]
*Thamnodynastes chaquensis (Bergna & Alvarez*,* 1993)*	*Raillietiella furcocerca*	NI	Crato, Ceará, Brazil	[10]
*Xenodon merremii* Wagler, 1824	*Porocephalus crotali*,* Sebekia sp.*	Trachea	NI	[30]
*X. merremii*	Raillietiella furcocerca*	Lung	Brazil	[51]
X. merremii*	*Raillietiella furcocerca*	NI	Bodoquena e Salobra, Mato Grosso, Brazil	[12]
*X. merremii*	*Raillietiella furcocerca*	Lung	Loma Plata, Paraguay	[46]
**Elapidae**				
*Micrurus* sp.	*Sebekia oxycephala* (nymph)*	Coelomic cavity	NI	[30]
*Micrurus fulvius* (Linnaeus, 1766)	*Kiricephalus coarctatus*	Nymphs and young adults in the muscles	Guatemala	[52]
*Micrurus ibiboboca* (Merrem, 1820)	*Raillietiella* sp. nov.	Lung	Crato, Ceará, Brazil	[9]
*Micrurus alleni* (Schmidt, 1936)	*Sebekia* sp. (nymphs)*	Coelomic cavity	Costa Rica	[53]
*Micrurus tener* (Baird & Girard, 1853)	*Kiricephalus coarctatus*	Lung and coelomic cavity	Gómez Farías, Tamaulipas, Mexico	[13]
*Micrurus surinamensis* (Cuvier, 1816)	*Sebekia oxycephala*	Coelomic cavity	Sinop, Mato Grosso, Brazil	[54]
**Viperidae**				
*Bothrops* sp.	*Porocephalus stilesi*	NI	Instituto Butantan, São Paulo, Brazil	[41]
*Bothropoides mattogrossensis* (Amaral, 1925)	*Raillietiella* sp.	Lung	Corumbá, Mato Grosso do Sul, Brazil	[10]
*Bothrops atrox* (Linnaeus, 1758)	*Raillietiella venteli*	Stomach	Porto Esperança, Mato Grosso do Sul, Brazil	[51]
*B. atrox*	*Kiricephalus* sp.	Nymph	Trinidad, West Indies	[43]
*B. atrox*	*Raillietiella venteli*	Stomach	Instituto Butantan, São Paulo, Brazil	[12]
*B. atrox*	*Raillietiella furcocerca*	NI	Trinidad, West Indies	[38]
*B. atrox*	*Porocephalus crotali*	Lung	Peruvian Amazon	[55]
***B. atrox***	***Raillietiella furcocerca***	**Lung and coelomic cavity**	**Santarém e Belterra, Pará, Brasil**	**Present study**
*Bothrops alternatus Duméril*,* Bibron & Duméril*,* 1854*	*Cephalobaena tetrapoda*	Lung	San Bernardinho, Paraguay	[56]
*B. alternatus*	*Porocephalus* cf. *crotali*	Lung	Route 17, Eldorado, Misiones, Argentina	[47]
*B. alternatus*	*Porocephalus crotali*	Lung	Northeastern Argentina	[34]
*Bothrops asper* (Garman, 1883)	*Porocephalus crotali*	Lung	Ocozocoautla, Chiapas, Mexico	^b^ [57, 71]
*B. asper*	*Porocephalus crotali*	Lung	San Juan Evangelista, Veracruz, Mexico	^b^ [57, 71]
*B. asper*	*Porocephalus crotali*	Lung	Sontecomapan, Veracruz, Mexico	^b^ [57, 71]
*B. asper*	*Porocephalus* cf. *clavatus*	Lung	Siquirres, Limón, Costa Rica	[16]
*B. asper*	*Kiricephalus coarctatus*	Lung	Gómez Farías, Tamaulipas, Mexico	[13]
*B. asper*	*Porocephalus stilesi*	Coelomic cavity	Gómez Farías, Tamaulipas, Mexico	[13]
*Bothrops diporus* Cope, 1862	Pentastomida indet.^a^	Lung	Northeastern Argentina	[34]
*Bothrops erythromelas* (Amaral, 1923)	*Cephalobaena tetrapoda*	Lung	Ceará, Brazil	[58]
*Bothrops jararacussu Lacerda 1884*	*Porocephalus stilesi*	Lung	South America	[41]
*B. jararacussu*	Pentastomida indet.^a^	Lung	Northeastern Argentina	[34]
*Bothrops jararaca* (Wied-Neuwied, 1824)	*Porocephalus crotali*,* Sebekia* sp.	Lung and coelomic cavity	Fazenda Real de Ipanema, Iperó, São Paulo, Brazil	[30]
*B. jararaca*	*Porocephalus stilesi*	Intestinal cavity	Fazenda Real de Ipanema, Iperó, São Paulo, Brazil	[30]
*B. jararaca*	*Sebekia oxycephala*	Lung	Fazenda Real de Ipanema, Iperó, São Paulo, Brazil	[30]
*B. jararaca*	*Porocephalus stilesi* (nymph)*	Encysted in the intestinal wall and liver	Fazenda Real de Ipanema, Iperó, São Paulo, Brazil	[30]
*B. jararaca*	*Porocephalus* sp.	Lung	Instituto Butantan, São Paulo, Brazil	[59]
*B. jararaca*	*Porocephalus stilesi*	Nymphs in the stomach and intestine	Instituto Butantan, São Paulo, Brazil	[41]
*Crotalus atrox* Baird & Girard, 1853	*Porocephalus crotali*	Lung	Ciénega de Flores, Nuevo León, Mexico	^b^ [57, 71]
*C. atrox*	*Porocephalus crotali*	Lung	Apodaca, Nuevo León, Mexico	^b^ [57, 71]
*Crotalus basiliscus* (Cope, 1864)	*Porocephalus crotali*	Lung	Colima, Mexico	^b^ [57, 71]
*Crotalus catalinensis* Cliff 1954	*Porocephalus crotali*	Lung	Ilha de Santa Catalina, Baja California, Mexico	[60]
*Crotalus durissus* Linnaeus, 1758	*Porocephalus crotali*	Lung	Zihuatanejo, Guerrero, Mexico	^b^ [57, 71]
*C. durissus*	*Porocephalus crotali*	Lung	El Mante, Tamaulipas, Mexico	^b^ [57, 71]
*C. durissus*	*Porocephalus crotali*	Lung and coelomic cavity	Mato Grosso, Brazil	[30]
*Crotalus durissus terrificus* Laurenti, 1768	*Porocephalus crotali*	Lung	Cumaná, Venezuela	[61]
*C. durissus terrificus*	*Porocephalus crotali*	NI	Instituto Butantan, São Paulo, Brazil	[56]
*C. durissus terrificus*	*Cephalobaena tetrapoda*	Lung	Instituto Butantan, São Paulo, Brazil	[12]
*C. durissus terrificus*	Pentastomida indet.^a^	Lung	Northeastern Argentina	[34]
*C. durissus terrificus*	*Porocephalus crotali*	Lung and air sac	Loma Plata, Paraguay	[46]
*C. durissus terrificus*	*Raillietiella furcocerca*	Stomach	Pirambóia, São Paulo, Brazil	[10]
*C. durissus terrificus*	*Porocephalus* sp.	Lung	Corumbá, Brazil	[10]
*C. durissus terrificus*	*Porocephalus* sp.	Lung	Minas Gerais, Brazil	[62]
*Crotalus tzabcan* Klauber, 1952	*Porocephalus crotali*	Lung	Felipe Carrillo Puerto, Península de Yucatán	[63]
*C. tzabcan*	*Porocephalus crotali*	Lung, coelomic cavity, liver and stomach	Calakmul, Campeche, Mexico	[64]
*C. tzabcan*	*Porocephalus crotali*	Lung	Chetumal, Quintana Roo, Mexico	[64]
*C. tzabcan*	*Porocephalus crotali*	Lung	Caobas, Quintana Roo, Mexico	[64]
*Metlapilcoatlus mexicanus* (Duméril, Bibron & Duméril, 1854)	*Porocephalus crotali*	Lung	Ocozocoautla de Espina, Chiapas, Mexico	^b^ [57, 71]
*M. mexicanus*	*Porocephalus crotali*	Lung	La Trinitaria, Chiapas, Mexico	^b^ [57, 71]
*M. mexicanus*	*Porocephalus crotali*	Lung	Tezonapa, Veracruz, Mexico	^b^ [57, 71]
*Lachesis sp.*	*Cephalobaena tetrapoda*	NI	San Bernardinho, Paraguay	[56]
*Lachesis sp.*	*Raillietiella furcocerca*	NI	Fazenda Taperinha, Santarém, Pará, Brazil	[12]
*Lachesis* (or *Bothrops*?) sp.	Cephalobaena tetrapoda	Lung	Loreto, Misiones, Argentina	[41]
*Lachesis rhombeata* (Wied, 1824)	*Porocephalus stilesi*	Lung	Pernambuco, Recife, Brazil	[65]
*Lachesis muta* (Linnaeus, 1766)	*Porocephalus stilesi*	NI	Puerto Viejo, Heredia, Costa Rica	[35]
*L. muta*	*Porocephalus stilesi*	Lung	Recife, Pernambuco, Brazil	[32]
*L. muta*	*Porocephalus stilesi*	Lung and coelomic cavity	Satipo, Departamento de Junín, Peru	[66]

*Palusophis bifossatus* (Raddi, 1820) (cited as *Coluber lichtensteinii* Wied-Neuwied, 1824 and *Dryadophis bifossatus* Raddi, 1820); *Mastigodryas bifossatus* (Raddi, 1820) (cited as *Coluber bifossatus* Raddi, 1820); *Boa imperator* Daudin, 1803 (cited as *Boa imperator constrictor*); *Eunectes notaeus* Cope, 1862 (cited as *Epicrates notaeus*); *Erythrolamprus poecilogyrus* (Wied-Neuwied, 1825) (cited as *Leimadophis poecilogyrus reticulatus*); *Erythrolamprus almadensis* (Wagler, 1824) (cited as *Leimadophis almadensis*); *Xenodon merremii* (Wagler, 1824) (cited as *Waglerophis merremii*); *Micrurus* sp. (cited as *Elaps* sp.); *Micrurus fulvius* (Linnaeus, 1766) (cited as *Elaps fulvius*); *Bothrops diporus* (Cope, 1862) (cited as *Bothrops neuwiedi diporus*); *Raillietiella furcocerca* (Diesing, 1835) (originally described as *Pentastoma furcocerca* Diesing, 1835); *Raillietiella gomesi* Motta, 1963, reported from *Xenodon merremii*, was subsequently placed in synonymy with *Raillietiella furcocerca*; *Raillietiella venteli* (Motta, 1965) (originally described as *Mahafaliella venteli* Motta, 1965; currently regarded as a *nomen dubium*); *Porocephalus crotali* (Humboldt, 1808) (originally described as *Echinorhynchus crotali* Humboldt, 1808 and subsequently cited as *Pentastoma proboscideum* Rudolphi by Diesing); *Porocephalus clavatus* (Wyman, 1845) (cited as *Pentastoma clavatum* Wyman, 1845), *Porocephalus clavatus* (Wyman, 1845) (reported as *Pentastoma imperatoris* Macalister, 1875 by Macalister, 1875).*Records reported as *Pentastoma gracile* by Diesing (1836) are treated individually because this name encompassed heterogeneous immature pentastomids subsequently assigned to different taxa. ^a^Pentastomida indet. was used for records from Martínez et al. (1999) for which the pentastomid taxon could not be reliably assigned at genus or species level to the respective host species. ^b^Records attributed to Peláez & Juliá (1983) were retrieved from Paredes-León et al. (2008), as the original publication was not directly examined

**Table 4:** Morphometric measurements (in milimeters) of *Raillietiella furcocerca* found in *Bothrops atrox* and other snakes in Brazil presented in Rego (1983)

Parameters of *Raillietiella furcocerca*	Hosts						
	*Xenodon merremii*		*Lachesis* sp.	*Drymarchon corais*		*Bothrops atrox* (present study)	
	M	F	F	M	F	M (*n* = 20)	F (*n* = 20)
Body length	18	72	52	18	85	16.5	14
Body width	1.48	3.50	3.40	2.00	0.4.40	1.90	0.19
Buccal complex length	0.25	0.46	0.60	0.33	0.55	0.27	0.26
Buccal complex width	0.10	0.20	0.34	0.18	0.31	0.195	0.20
Anterior hook blade	0.09	0.20	0.20	0.09	0.46	0.09	0.095
Complete anterior hook	0.25	0.44	0.50	0.29	0.56	0.28	0.27
Posterior hook blade	0.12	0.31	0.32	0.48	0.86	0.27	0.27
Complete posterior hook	0.41	0.92	0.90	0.19	0.33	0.62	0.59
Base width of RCD	–	–	–	–	–	0.41	–
Total length of RCD	–	–	–	–	–	0.95	–
Apical width of RDC	–	–	–	–	–	0.027	–
Base width of LDC	–	–	–	–	–	0.31	–
Total length of LCD	–	–	–	–	–	0.98	–
Apical width of LCD	–	–	–	–	–	0.040	–

RCD: right copulatory dilators, LCD: left copulatory dilators, M: male, and F: female

## Discussion

In this study, the first record of *R. furcocerca* in *B. atrox* from the Amazon region expands the known geographic distribution of this parasite in Brazil. Furthermore, the recovery of specimens from the coelomic cavity represents a relevant finding, given that pentastomids are classically considered obligate parasites of the vertebrate respiratory system [10–12]. Extrapulmonary infections by pentastomids have already been documented in Neotropical snakes involving *Porocephalus* sp. and *S. oxycephala* [10, 17, 54, 62, 67], as well as species of the same genus identified here, including *R. venteli* and *R. furcocerca* [12, 17]. The presence of adult pentastomids in extrapulmonary sites such as the stomach, liver, and coelomic cavity raises hypotheses regarding active tissue migration or variations in the parasite’s life cycle, suggesting that ecological and behavioral aspects of the host, as well as environmental factors, may influence nymph dispersion and attachment in the hosts. However, interpretation of extrapulmonary localization requires caution and further studies are needed.

*Post-mortem* displacement is a well-documented phenomenon in helminths and pentastomes, wherein parasites migrate passively or actively through tissues following host death, occasionally emerging into coelomic or oral cavities [68, 69]. In our study, the detection of pentastomids actively attached to the visceral tissues, coupled with localized hemorrhagic tissue reactions, supports true antemortem extrapulmonary localization rather than artifactual post-mortem displacement.

The overall prevalence of *R. furcocerca* observed (18.8%) was higher than that reported by Carvalho et al. (2018) [14] in *L. annulata* in Ceará, northeastern Brazil, but lower than the prevalence reported by Almeida et al. (2007) [9], who found a higher prevalence (35%) in *P. nattereri* in the municipality of Crato, Ceará, Brazil. Here, in this study in the Lower Amazon region, we present new data on the prevalence, intensity, and mean abundance of *R. furcocerca* infection in *B. atrox*. This information contributes to the understanding of the ecological relationships between this parasite and its respective host.

Previous studies restricted pentastome records primarily to hosts from the Atlantic Forest and Caatinga biomes [21]. Our literature review demonstrates that *R. furcocerca* possesses a broad geographic range across the Amazon, Caatinga, and Atlantic Forest biomes, infecting a wide diversity of snake hosts. The present finding confirms that *B. atrox* serves as a suitable host for *R. furcocerca* in the Amazonian biome, expanding our understanding of the geographic and host ranges of these pentastomids in South America.

Patterns emerging from our review demonstrate a high frequency of records for the family Porocephalidae Sambon, 1922 (18 interaction records encompassing 5 species across 2 genera: *Porocephalus crotali*, *P. clavatus*, *P. stilesi*, and *Kiricephalus coarctatus*) and Raillietiellidae Sambon, 1922 (24 records encompassing 3 species: *R. furcocerca*,) in Neotropical snakes. Rather than indicating absolute biological dominance across ecosystems, this taxonomic pattern primarily reflects historical differences in research effort and parasite detectability across regions and host groups. Similarly, the wide host spectrum reported for *Raillietiella* is directly associated with the high concentration of parasitological surveys targeting this genus, showing that increased sampling effort expands known host associations.

Although molecular and phylogenetic data for Pentastomida have expanded globally in public databases a pronounced sequence gap persists for Neotropical snake parasites. Among the 11 pentastomid species identified in our synthesis of Neotropical snakes, only 3 species (*Porocephalus crotali*, *P. clavatus*, *S. oxycephala*) have published sequence data (mainly nuclear 18 S/28S rDNA and mitochondrial * COX1*/* 12S* rDNA). Crucially, the majority of these sequences derive from Central/North American or imported captive specimens. Widespread Neotropical taxa endemic to or predominant in South America, most notably *R. furcocerca* (* n=12* host records) and *C. tetrapoda* (* n=7* host records), currently lack any deposited molecular sequence data. Because pentastomids exhibit conservative morphological characters, the reliance on physical identification alone leaves current estimates of species richness, geographic range, and host specificity vulnerable to underestimating cryptic diversity. Integrating molecular tools (such as DNA barcoding and multi-locus phylogenetics) with classical morphometrics is imperative to test whether widespread lineages like *R. furcocerca* represent single generalist species or complexes of cryptic taxa. The marked geographic concentration of studies in northeastern and southeastern Brazil reveals significant sampling gaps in other South American ecosystems. Although Brazil encompasses more than 60% of the Amazonian biome, existing Brazilian surveys are heavily concentrated in the Caatinga and Atlantic Forest biomas, leaving the vast Brazilian Amazon as well as neighboring Amazonian territories (e.g., Peru, Colombia, Venezuela) severely underrepresented [12, 13, 15, 17, 21, 22, 53, 54, 63, 64, 66, 67, 70]. These geographic discrepancies stem from logistic challenges in remote field sampling and wild host handling. As a result, pentastomids infecting wild Amazonian snakes remain poorly studied compared to parasites of medical or veterinary concern. Future research must prioritize targeted field surveys combined with molecular characterization of pentastomids across underexplored Amazonian habitats to unravel their true evolutionary diversity and transmission pathways.

## Data Availability

No datasets were generated or analysed during the current study.
